# Readiness as a precursor of early implementation outcomes: an exploratory study in specialty clinics

**DOI:** 10.1186/s43058-022-00336-9

**Published:** 2022-09-03

**Authors:** Melanie Livet, Carrie Blanchard, Chloe Richard

**Affiliations:** Center for Medication Optimization (CMO), Division of Practice Advancement and Clinical Education, UNC Eshelman School of Pharmacy, University of North Carolina at Chapel Hill, Chapel Hill, NC USA

**Keywords:** Readiness, Implementation outcomes, Acceptability, Appropriateness, Feasibility, Intent to adopt, Pharmacy practice

## Abstract

**Background:**

Readiness has been identified as an essential precursor of successful implementation. However, evidence supporting its value is sparse. Empirical studies exploring the relationship between the application of readiness interventions, readiness levels, and implementation outcomes are lacking. The purpose of this study was twofold: (1) to evaluate the effectiveness of a readiness intervention (based on increases in readiness levels, changes in early implementation outcomes (i.e., acceptability, appropriateness, feasibility, and intent to adopt), and qualitative insights into the types of perceived outcomes) and (2) to assess the role of readiness as a predictor of these early implementation outcomes.

**Methods:**

Seven healthcare specialty clinics engaged in a structured process to assess and build readiness for implementing a comprehensive medication management (CMM) service over a 10-month period. A mixed methods approach, which included surveys with healthcare stakeholders at each clinic (*N* = 27) and interviews with the lead pharmacists (*N* = 7), was used to evaluate the effectiveness of the readiness intervention (aim 1). Survey data were also used to conduct multiple regression models to assess the role of readiness as a predictor of early acceptability, appropriateness, feasibility, and intent to adopt CMM (aim 2).

**Results:**

Significantly higher readiness levels, as well as higher scores on acceptability, appropriateness, feasibility, and intent to adopt, were reported as a result of engaging in the readiness intervention. However, upon closer examination, the direction of this association seemed to be dependent on the type of clinic. Qualitative data on the types of perceived outcomes resulting from engaging in the readiness intervention provided further insights into the potential reasons for these findings. Furthermore, post-readiness levels predicted between 44 and 68% of the variance in the early implementation outcomes. When accounting for clinic membership, readiness remained critical for service acceptability, feasibility, and intent to adopt but not for appropriateness.

**Conclusion:**

These findings provide insights into the relationship between use of a readiness intervention, readiness levels, and early implementation outcomes. Engaging healthcare settings in a readiness intervention was beneficial in ways more complex than a simple positive linear relationship, highlighting the opportunity to broaden its purpose and expand definitions of readiness success. In addition, the importance of readiness levels in predicting early implementation outcomes, while critical, also seems to be highly dependent on context, particularly for appropriateness (fit).

Contributions to the literature

- Although readiness has been identified as an essential precursor of implementation, the empirical evidence supporting the linkages between readiness and implementation is sparse.
- This is the first published study to explore the relationship between the application of a readiness intervention, grounded in the R=MC^2^ heuristic, readiness levels, and early implementation outcomes.
- Based on these findings, the relationship between readiness and implementation is significant but more complex than a simple positive linear relationship, with the readiness intervention particularly beneficial for clinics that determine service fit prior to participation.

## Introduction

Establishing readiness has been identified as a critical step in the implementation process [1–6]. To deliver a new service, intervention, program, technology, or policy, a healthcare organization needs to be first and foremost *willing* (e.g., committed) and *able* (e.g., capable) to embrace this change [1, 6–8]. Lack of readiness has been assumed to lead to failed implementation and, ultimately, decreased likelihood of achieving the health outcomes typically associated with a service. In fact, it has been suggested that one-half of all unsuccessful, large-scale organizational change effort could be attributed to a lack of readiness [6, 9].

Although recommendations to prepare an organization for implementation align with the organizational change literature, efforts to empirically demonstrate the relationship between use of a readiness intervention, readiness levels, and implementation outcomes have been limited. Existing studies typically focus on specific aspects of readiness (e.g., capacity-building strategies) and their influence on implementation success, rather than evaluate a comprehensive readiness intervention [10, 11]. The lack of data could be attributed to a number of reasons, including the sparsity of applicable readiness frameworks [12], limited opportunities for investigating readiness as an applied intervention [13–15], and lack of adequate readiness measures [6, 16–18]. The need for evidence to support the theoretical linkages between readiness and implementation outcomes has repeatedly been emphasized by several groups of researchers [6, 12, 14].

The current study contributes to this gap by exploring the relationship between use of a readiness intervention (informed by the R=MC^2^ heuristic) [12], self-reported readiness levels (i.e., commitment and ability to implement a new service) [6], and early implementation outcomes (i.e., feasibility, appropriateness, acceptability, and intent to adopt) [19]. This evaluation was conducted as part of a broader initiative designed to assess the feasibility of implementing a new service, comprehensive medication management (CMM), in specialty clinics (e.g., melanoma, bone marrow transplant) located within a large, academic medical center.

CMM is a patient-centered clinical service delivered by pharmacists in collaboration with other health care team members to maximize a patient’s medication regimen and improve health outcomes [20]. This care process ensures that patients’ medications are individually evaluated to determine appropriateness, effectiveness, safety given concurrent therapies, and feasibility to take as intended. The pharmacist is responsible for developing and managing an individualized medication therapy care plan in collaboration with the patient and the healthcare team, including appropriate follow-up to ensure optimal medication use. This intervention has been clearly defined elsewhere through the articulation of key principles, essential functions, and operational definitions [21, 22].

To prepare for implementation, participating clinics engaged in a four-phase readiness intervention (i.e., a structured process to assess and build readiness for implementation, detailed below) over a 10-month period. The readiness intervention, designed to increase both the *commitment* and *capabilities* to implement CMM, was grounded in the R=MC^2^ heuristic [12]. Although readiness is a concept included in some implementation models, efforts to translate it into a usable intervention are severely lacking [2, 5–7, 18, 23, 24]. R=MC^2^ was selected for this study as the only applied readiness assessment and building process with existing tools, resources, and practices.

Briefly, R=MC^2^ defines readiness as a multi-faceted concept that results from the interplay of three components: motivation (i.e., perceived incentives and disincentives that contribute to the desirability of an innovation), general capacity (i.e., conditions related to how well an organization is functioning), and innovation-specific capacity (i.e., conditions needed to implement a specific innovation). Each of these components was further defined into a number of subcomponents described elsewhere [15] (e.g., champions, supportive climate, innovation-specific knowledge and skills, and interorganizational relationships). R=MC^2^ was initially created and translated by Wandersman and colleagues into a set of practical tools and practices (such as a Readiness Diagnostic Scale and the use of heatmaps). The heuristic was further operationalized into a readiness intervention by the lead author and later refined in collaboration with the Wandersman group [14, 15].

R=MC^2^ was developed as one element of the Interactive Systems Framework (ISF) [25]. The ISF is designed to bridge the gap between science and practice and depicts the basic structure of an implementation system. It involves three interconnected systems (i.e., the prevention synthesis and translation system, the support system, and the delivery system) that work together to facilitate implementation and dissemination of innovations. It has been widely used in a number of fields, including public health, community, education, and healthcare, and is described elsewhere [25]. Assessing and building readiness within the ISF is a function of the support system (i.e., individuals and settings responsible for capacity building of the delivery system through technical assistance, training, and other strategies) to prepare the delivery system (i.e., individuals and settings involved in the delivery of the innovation) for implementation.

The aims of this exploratory study were to (1) evaluate the effectiveness of the readiness intervention and (2) assess the role of readiness as a predictor of early implementation outcomes. These findings provide preliminary insights into the role, value, and impact of a readiness intervention, as well as data on the relationship between readiness levels and early implementation outcomes. To ascertain the importance of readiness in applied implementation science, it is critical for it to be subject to more extensive empirical study.

## Methods

### Study design overview

This exploratory study used mixed data collected as part of the broader initiative described above. The effectiveness of the readiness intervention (aim 1) was evaluated based on the quantitative changes in readiness levels and early implementation outcomes scores (i.e., acceptability, appropriateness, feasibility, and intent to adopt) from baseline to post-readiness intervention. In addition, the types of outcomes experienced by the pharmacists as a result of participating in the readiness intervention were explored qualitatively using interview data. The aim 1 analysis employed a sequential mixed methods balanced structure for the purpose of seeking complementarity (i.e., for answering different aspects of the same overall question), with the qualitative dataset embedded into the quantitative study [26].

The role of readiness as a predictor of early implementation outcomes (aim 2) was assessed through a series of regression models using post-readiness levels (readiness scores following the readiness intervention) as predictors and post-implementation outcomes scores as dependent variables. These implementation outcomes (Table 1) were selected as early indicators of perceived implementation success based on the implementation stage that participating sites were engaged in at the time of data collection (i.e., pilot testing of a new service).

**Table 1:** Implementation outcomes definitions

Implementation outcome	Definition
Acceptability	Perceptions that the service is agreeable, palatable, or satisfactory
Appropriateness	Perceived fit, relevance, or compatibility of the service for a given practice site, provider, or consumer and/or perceived fit of the innovation to address a particular issue or problem
Feasibility	Extent to which a service can be successfully used or carried out within a given practice site (suitability or practicability)
Intent to Adopt	The intention, initial decision, or action to try or use the service (uptake, utilization, initial implementation, intent to try)

Data sources included surveys with members of the implementation team at each clinic, including the lead pharmacists (*N* = 27), and interviews with the lead pharmacists (*N* = 7). All data were collected at the end of the readiness intervention period as the clinics were transitioning to pilot testing of CMM. The retrospective pre-post survey method was used for all surveys to control for response-shift bias [27]. Informed consent was collected prior to participant engagement for both the survey and interviews. All participants were offered a small gift card incentive for completing the survey. Participation in the interviews was not compensated.

### Setting, recruitment, and expectations

A total of eight healthcare specialty clinics within the academic medical center were initially recruited to participate in this project. One of the clinics dropped out shortly after the beginning of the readiness intervention due to competing priorities and an unforeseen leave of absence of the lead pharmacist. Table 2 describes the remaining seven clinics in more detail. The number of clinics was dictated by the project scope and requirements for patient enrollment numbers as part of the broader initiative. The clinics were selected by the research group in collaboration with the Medical Center Department of Pharmacy (MCDOP) managers based on the following criteria: clinic pharmacist’s bandwidth for implementing a new service, potential for involving other members of the healthcare team at this clinic, and level of interest in pilot testing CMM. Once the pharmacists expressed interest, they were provided with a written description of the project and an overview of expectations (including readiness activities and deliverables, number of expected participation hours, and a timeline). The research group worked very closely with the MCDOP managers over the course of the project, meeting monthly, to discuss progress, problem solve as needed, and review any improvements or modifications to the process.

**Table 2:** Description of participating clinics

Clinic type (focus area)	Unique number of patients served	Total number of clinical pharmacists	Total number of physicians	Number of implementation team members (including the pharmacists)
Bone marrow transplant	712	2	7	7
Genitourinary oncology	1074	1	5	4
Heart and vascular	9790	4	44	4
Melanoma	1686	1	7	2
Myeloma	948	1	3	4
Neurology	9704	1	40	3
Pain	1087	1	3	4

### The readiness intervention

**Fig. 1:**
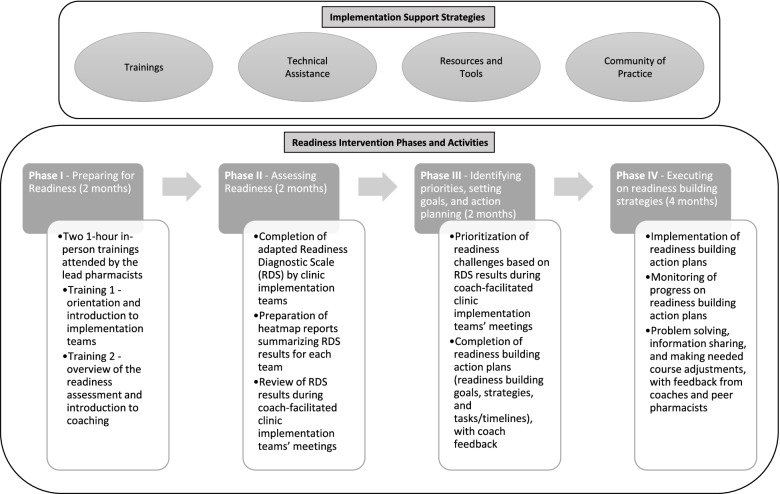
Overview of the readiness intervention

The readiness intervention was organized into four phases: preparing for readiness (2 months); assessing readiness (2 months); identifying priorities, setting goals, and action planning (2 months); and readiness building strategy execution (4 months). Figure 1 provides an overview of the readiness intervention. During phase I, *preparing for readiness*, lead pharmacists attended two in-person 1-h trainings delivered by the research group. The first training was designed to introduce the pharmacists to the readiness intervention, further describe the service they were interested in adopting (CMM), review expectations of participation, and support the building of implementation teams (involving 2–7 clinic team members). Of note, while the expectation was that the lead pharmacists engage in all of the readiness activities, the involvement of other clinic members was left at the discretion of the pharmacists. The second training provided an overview of the readiness assessment process and introduced the pharmacists to their respective coaches.

In phase II, *assessing readiness*, lead pharmacists and members of their implementation team completed an adapted version of the Readiness Diagnostic Scale (RDS) individually [13, 14]. The adapted RDS is an 88-item Likert scale tool designed to assess readiness strengths and areas for improvement across the three R=MC^2^ components (motivation, general capacity, and innovation-specific capacity). The results from the RDS were then compiled into heatmap reports tailored to each team. These reports summarized the results from the RDS, highlighted the readiness strengths and challenges, and listed the key insights reported by the team members. These reports were reviewed by the teams and discussed with the coaches during their regularly scheduled check-in calls.

In phase III, *identifying priorities, setting goals, and action planning*, each team prioritized their readiness challenges based on their readiness assessment results. They were also asked to build action plans outlining their readiness goals (e.g., to increase knowledge and awareness of CMM among clinic team members), readiness building strategies (e.g., conduct a training session), specific tasks (e.g., develop a slide deck to present to team members), timeline for completion, and person(s) responsible. The coaches assisted each team with the prioritization of challenges using a four-quadrant priority matrix (low-high on feasibility and on impact of the service). They also provided the teams with an action plan template and provided feedback once the plans were completed.

During the final phase, *readiness building strategy execution*, teams executed their previously developed action plans, tracked their progress, and made adjustments as necessary. Of note, although the readiness action plans were individualized to each clinic, with each lead pharmacist deciding the readiness priority areas to focus on for their clinic, commonalities were quickly noted by the project team (e.g., planning for workflow integration, gaining buy-in from other clinic stakeholders).

In addition to the trainings described above, the implementation support strategies for this project included (1) monthly community of practice calls to facilitate information sharing between the lead pharmacists and the research group, encourage peer networking and discussions, provide a forum for participants to problem-solve and support each other in this work, and address the common readiness areas of focus noted above; (2) development and provision of guidance and tools to facilitate team building, completion of the readiness assessment, prioritization of challenges, strategy identification, and action planning; and (3) provision of individualized assistance by three members of the research group serving as coaches.

The coaches were identified based on previous experience with the readiness intervention and/or previous experience with coaching pharmacists. The clinics had access to their coaches through regularly scheduled meetings and via email and phone as needed. The lead pharmacist served as the point person for their teams, with involvement of other team members at their discretion. A standardized coaching process was developed as part of the project for all of the participating clinics, with coaching agendas created ahead of time for each phase of the readiness intervention. The coaches recorded notes and progress in a standardized coaching log, which was updated following any contact with the clinics, with monthly internal meetings serving to review updates and discuss any challenges. Fidelity to the readiness intervention was ensured through these activities as well as standardization of readiness activities across sites, weekly research group project check-ins, and review of coaching logs.

### Data collection

#### Surveys

Surveys included demographics questions, an adapted version of the Organizational Readiness for Implementing Change (ORIC) [18] to assess readiness levels, and the Implementation Outcomes Questionnaire (IOQ) [28] measures to assess acceptability, appropriateness, feasibility, and intent to adopt. The demographics questions included gender, race, degree, age, and professional role, as well as years of professional experience, years working at this particular clinic, level of involvement in the readiness intervention, and clinic membership (used interchangeably with clinic type). The level of involvement was assessed according to the number of readiness activities the respondent participated in, with response categories ranging from “not at all,” to “somewhat (at least one of the above activities),” to “fully.” The readiness activities (e.g., in-person trainings, development of CMM readiness action plan, progress monitoring) were listed for reference in the survey. Items from the previously validated ORIC were tailored to assess individual (rather than organizational) ability and motivation to implement CMM (i.e., “I” instead of “people who work here,” “CMM” instead of “this change”). The retrospective pre-post implementation outcome measures from the previously validated IOQ are described in Table 3.

**Table 3:** IOQ implementation outcomes measures

Outcome measures	Number of items	Likert scale	Reliability (Cronbach’s alpha)		Example item
			Pre	Post	
Acceptability	7	6-point (strongly disagree–strongly agree)	0.91	0.90	I am in favor of delivering CMM.
Appropriateness	6	5-point (not at all–extremely)	0.86	0.92	How well does CMM fit with the clinic’s overall approach to patient care?
Feasibility	8	6-point (strongly disagree–strongly agree)	0.92	0.92	The healthcare professionals and staff needed to carry out CMM were reasonable.
Intent to adopt	2^a^	6-point (strongly disagree–strongly agree)	0.97	0.95	I plan to use CMM.

^a^Two items from the original measure were included in the analyses. The other three items were not included due to the focus on the decision to adopt rather than adoption facilitators. Due to the nature of the readiness intervention (e.g., participants were expected to attend trainings), scoring on these three items was not expected to differ across participants

#### Interviews

Thirty-minute semi-structured interviews were conducted with the lead pharmacists over a 2-week period by a research group member. The interviewer had no previous interactions with the clinics thereby minimizing unduly influence on participant responses. The interviews, conducted as part of the broader initiative, focused broadly on the participants’ experiences with the readiness intervention (e.g., successes, challenges, lessons learned, results). However, this specific study only made use of data coded as “result” since its focus was to better understand the perceived outcomes resulting from participation in the readiness intervention (see the “Data analysis” section). Interview questions (e.g., Could you please describe some of the changes that resulted from engaging in the readiness intervention? How has the readiness intervention changed your beliefs about the implementation of CMM in your clinic, if at all?) were developed ahead of time to standardize the interviews. During the interviews, participants were first reminded of the specific activities that were part of the readiness intervention (e.g., completing the readiness assessment, identifying priorities, action planning) to ensure shared understanding. With consent from each participant, all interviews were recorded and transcribed to facilitate analyses.

### Data analysis

#### Evaluating the effectiveness of the readiness intervention (aim 1)

Descriptive statistics were initially computed in all surveys. Paired samples *t*-tests were run to compare the pre- and post-intervention scores for readiness levels, feasibility, and intent to adopt. Wilcoxon rank tests were conducted with acceptability and appropriateness due to violation of the normality assumption based on a review of the Shapiro-Wilks test. To assess whether clinic membership impacted post-intervention readiness levels and implementation outcomes when controlling for pre-scores^1^, two-way mixed ANOVAs were calculated with the appropriate pre-score as the within-variable and clinic membership as the between-variable. For acceptability, appropriateness, and intent to adopt, Scheirer-Ray-Hare (rank-order ANOVAs) tests were also used to confirm the results from the two-way mixed ANOVAs because of mild non-normality and homogeneity issues with the data. Because the results were similar regardless of the statistical methods, only the results from the two-way mixed ANOVAs are reported below.

Interview transcript data were examined using directive content analysis [29]. During the pre-coding stage, the analyst read the transcripts to become familiar with the material. With the goal for this manuscript being to understand the types of perceived outcomes resulting from engaging in the readiness intervention, the analyst then coded the interview transcripts for “results.” Secondary codes were inductively identified as a result of this second read (e.g., increased understanding of CMM components, increased understanding of the patient population that CMM would benefit most, increased buy-in). The codebook was refined to include the primary deductive and secondary inductive codes. The third read involved the final coding of the transcripts. This information was transferred from the transcripts into a word document that organized the relevant pieces of texts into codes and subcodes. This information was then grouped into the themes reported in this manuscript. The analysis was conducted by the third author and reviewed by the first author. Disagreements were reviewed until consensus was reached.

#### Readiness levels as predictors of early implementation outcomes (aim 2)

Single and multiple linear regressions were conducted to assess the predictive strength of readiness levels for each of the early implementation outcomes (acceptability, appropriateness, feasibility, and intent to adopt). The initial models included post-intervention readiness scores as the independent variable (IV) and each of the post-intervention implementation outcomes as the dependent variable (DV). The second set of models included other significant IVs as guided by correlational results (between each of the DVs and each of the following IVs: years of professional experience, years working at the clinic, level of involvement in the readiness intervention, and clinic membership). Pearson’s bivariate correlations were used to assess the relationship between the first three IVs and each of the DVs. The eta coefficient was used to estimate the strength of association between clinic membership and the DVs. *R*-squared estimates are reported as indicators of the variance accounted for by the IV for each model. Beta weights (standardized multiple regression coefficients) and uniqueness indices were examined as estimates of the relative importance of each IV in the second set of models. Uniqueness indices were obtained by calculating the *R*-squared differences between each of the original regression models and each of the restricted or nested models. Regression assumptions (normality, homoscedasticity, linearity) were verified prior to analyses. In addition, multicollinearity checks included a review of two diagnostic indices, the tolerance and variance inflation factor (VIF) (i.e., reciprocal of its tolerance; it measures how much the variance of the estimated regression coefficients is inflated when compared to having uncorrelated predictors). None of the models had tolerance values below .20 and VIFs greater than 5. All analyses were conducted using IBM SPSS Statistics v26.

## Results

### Study sample

The participant sample for the current study included 27 healthcare professionals (out of the potential 28 implementation team members; see Table 2) from seven specialty clinics within one medical system. Thirty-seven percent (*N* = 10) of participating clinic members were pharmacists, 29.6% (*N* = 8) were nurses, 14.8% (*N* = 4) were physicians, 14.8% (*N* = 4) were advanced providers (PA, NP), and one was an administrator. The sample was predominantly female (70.4%) with a racial distribution of 96.3% white. Almost half (48.1%) had advanced medical or graduate degrees (PharmD, MD, PhD) with the average number of years of professional experience being 13.4 (SD = 7.9) and average length of working in their particular clinic being 4 years (SD = 2.9). Sixty-three percent (*N* = 17) reported being somewhat involved with the readiness intervention (i.e., completing at least one readiness activity as defined in the survey), 29.6% (*N* = 8) being fully engaged (including all of the lead pharmacists), and 7.4% not really being involved (*N* = 2).

### Was the readiness intervention effective?

#### Changes in readiness levels

On average, participants reported significantly higher levels of readiness from pre- to post-intervention (*t*(24) = 3.37, *p* = .00) (Table 4). Based on the results of the two-way mixed ANOVA, there was a significant interaction between clinic membership and readiness levels over time, *F*(6, 18) = 3.39, *p* = .02, partial *η*^2^ = .53. Readiness scores for five of the clinics increased over time, while two of the clinics reported lower post- compared to pre-intervention readiness levels (clinic A pre-score: *M* = 2.69, SD = .55; clinic A post-score: *M* = 2.31, SD = .39; clinic B pre-score: *M* = 3.33, *SD* = .48; clinic B post-score: *M* = 2.90, *SD* = .34).

**Table 4:** Readiness scores by clinic

Clinic	Readiness–efficacy, mean (SD)		Readiness–commitment, mean (SD)		Readiness–overall, mean (SD)	
	Pre	Post	Pre	Post	Pre	Post
A	2.62 (1.51)	2.05 (1.07)	2.80 (1.56)	2.67 (1.47)	2.69 (1.51)	2.31 (1.22)
B	3.54 (0.89)	3.11 (0.82)	3.05 (0.25)	2.60 (1.00)	3.33 (0.61)	2.90 (0.44)
C	2.71 (0.86)	3.82 (0.63)	3.55 (1.11)	3.90 (0.74)	3.06 (0.92)	3.85 (0.63)
D	2.71 (0.86)	4.12 (0.38)	2.83 (1.39)	4.33 (0.69)	2.76 (1.02)	4.21 (0.44)
E	2.95 (0.72)	3.95 (0.79)	3.00 (1.00)	4.27 (0.64)	2.97 (0.83)	4.08 (0.72)
F	2.05 (1.00)	3.76 (0.79)	2.40 (0.72)	3.93 (0.90)	2.19 (0.88)	3.83 (0.83)
G	3.14 (0.61)	3.86 (0.20)	3.70 (0.14)	4.20 (0.57)	3.38 (0.29)	4.00 (0.35)
All clinics	2.82 (0.93)	3.58 (0.90)	3.02 (1.02)	3.72 (1.00)	2.90 (0.90)	3.63 (0.89)

#### Changes in early implementation outcomes

Table 5 presents pre- and post-scores for acceptability, appropriateness, feasibility, and intent to adopt. Based on the paired *t*-test results for feasibility and adoption and results from the Wilcoxon rank test for appropriateness and acceptability, scores increased significantly from pre- to post-intervention for all of the implementation outcomes (feasibility: *t*(24) = 2.88, *p* < .01; adoption: *t*(24) = 6.08, *p* < .01; appropriateness: *z* = 3.28, *p* < .01; and acceptability: *z* = 1.98, *p* < .05).

**Table 5:** Early implementation outcomes scores by clinic

Clinic	Acceptability, mean (SD)		Appropriateness, mean (SD)		Feasibility, mean (SD)		Intent to adopt, mean (SD)	
	Pre	Post	Pre	Post	Pre	Post	Pre	Post
A	4.48 (0.54)	4.05 (0.73)	2.83 (0.58)	3.00 (0.60)	2.92 (1.23)	2.63 (1.07)	1.67 (0.58)	3.00 (1.73)
B	4.25 (0.59)	3.82 (0.49)	2.08 (0.29)	1.96 (0.16)	4.03 (0.86)	3.38 (0.62)	2.50 (1.29)	2.50 (0.58)
C	4.54 (0.61)	5.11 (0.21)	3.21 (0.16)	3.58 (0.17)	3.41 (0.98)	4.03 (0.77)	3.50 (1.29)	4.25 (0.50)
D	5.19 (1.00)	5.60 (0.58)	4.08 (0.96)	4.47 (0.50)	3.31 (0.52)	4.73 (0.57)	2.17 (1.47)	5.33 (0.52)
E	4.52 (0.60)	5.43 (0.52)	3.33 (0.50)	4.22 (0.63)	2.79 (0.80)	4.75 (0.54)	3.00 (1.73)	5.33 (0.52)
F	4.38 (0.50)	5.00 (0.00)	3.22 (0.92)	3.56 (0.51)	3.79 (1.73)	4.75 (0.45)	1.67 (0.58)	4.33 (1.16)
G	4.71 (0.40)	4.93 (0.10)	3.00 (0.24)	3.50 (0.00)	3.56 (0.80)	4.13 (0.18)	1.50 (0.71)	5.00 (0.00)
All clinics	4.63 (0.70)	4.90 (0.79)	3.19 (0.87)	3.53 (0.93)	3.41 (0.94)	4.11 (0.94)	2.36 (1.29)	4.28 (1.31)

The results from the two-way mixed ANOVAs highlighted significant interactions between clinic membership and two implementation outcomes over time, feasibility (*F*(6, 18) = 3.35, *p* = .02, partial *η*^2^ = .53) and intent to adopt (*F*(6, 18) = 4.77, *p* < .01, partial *η*^2^ = .61). Similarly to the readiness levels results above, feasibility scores increased over time for five of the clinics, with the same two clinics (clinics A and B) reporting decreased feasibility (Table 5). In addition, clinic A and clinic B were the only two clinics still reporting various levels of disagreement (*M* < 3.5) in intent to adopt post-intervention.

For acceptability and appropriateness, there was a main effect of clinic membership for acceptability (*F*(6, 18) = 3.65, *p* = .02, partial *η*^2^ = .55) and main effects of both time and clinic membership for appropriateness (time: *F*(1, 18) = 20.00, *p* < .00, partial *η*^2^ = .53; clinic membership: (*F*(6, 18) = 8.07, *p* < .00, partial *η*^2^ = .73). It is worth noting that examination of profile plots demonstrated that acceptability scores for clinic A and clinic B and appropriateness scores for clinic B were trending down, while all of the other clinics demonstrated increases in scores. Of note, the appropriateness for clinic A was also lower than that for the other clinics, with minimal movement from pre- to post-intervention.

#### Types of perceived outcomes from engaging in the readiness intervention

Based on the lead pharmacists’ interview data, perceived outcomes were grouped into the following themes: an improved understanding of the service, facilitated buy-in and engagement, additional insights into the feasibility of CMM, and an increased appreciation for the value of readiness.

##### Improved understanding of the service

Six of the interviewees noted that both the pharmacists and other clinic stakeholders had gained an improved understanding of the service as a result of the readiness intervention (e.g., “I think it brought CMM awareness to my team members, because my team members were [a] physician assistant and a nurse”). They reported a better grasp of the service components (e.g., “I think we learned a lot more about CMM and what it all entails”), as well as a refined ability to identify the population that would benefit most from the service (e.g., “We saw how other places implemented it and that it was a more selective process in identifying which patient groups within our clinics we wanted to offer CMM to”).

##### Facilitated buy-in, engagement, and relationship building

In addition, the readiness intervention served to deepen the buy-in and engagement of diverse clinic stakeholders (e.g., “We got a really good buy-in, not just from physicians but also our advanced practice providers and nursing staff”). One interviewee highlighted conversations with colleagues about the need to engage multiple disciplines to facilitate the integration of CMM. Another noted that having to engage others within the clinic had strengthened relationships and led to a greater appreciation of everyone’s roles and responsibilities.

##### Produced insights into the feasibility of CMM for specialty clinics

By engaging in the readiness intervention, the lead pharmacists gained additional insights into the feasibility of implementing CMM within their respective clinics (e.g., “A lot of my thoughts about the CMM process, specifically for specialty, revolve around how you identify what CMM means in a specialty clinic. So I think taking the think time to really figure out what works best for your clinic, what medicines are you going to address and is that still considered CMM”). Per the interviewees, these learnings influenced their decision to adopt the service. Those who decided against implementation identified insufficient time and resources and, more importantly, lack of service fit with clinic priorities as critical challenges (e.g., “I guess I learned that it’s not a good fit, that there were more barriers than I anticipated and that it wasn’t really in line with the goals of our clinic was really what it came down to”).

##### Increased appreciation for the readiness intervention and its benefits

Interviewees also expressed a deeper appreciation for the importance of preparing for implementation in a systematic and organized way. Engaging in the readiness intervention helped demystify the preparation process (e.g., “The process to me for readiness was really cool. I’ve never been involved in a readiness type of initiative before”); facilitated the generation and organization of ideas (e.g., “So I think a lot of that idea generation that occurred, especially in our meetings with our coach, really brought those things to the forefront made planning actionable”); helped identify and address potential implementation barriers (e.g., “But then the survey helped kind of narrow down those barriers”); stimulated the creation of actionable plans (e.g., “and we were able to develop more specific plans and assess our readiness more appropriately for that”); promoted accountability (e.g., “It was good to keep me on track”); and even provided a reason to discuss changes at the clinics (e.g., “It was nice for those pharmacists or those CPPs [clinical pharmacist practitioners] who maybe want to change up services they offer...kind of a natural way to discuss with their team what they want their services to look like,” “What we identified as we were going through some of our processes is that there was lots of redundancies happening for certain patient visits, especially our transition of care visits”).

### Were readiness levels predictive of early implementation outcomes?

Years of professional experience, years working at the clinic, and level of involvement in the readiness intervention were not significantly correlated with any of the early implementation outcomes based on Pearson’s r results. Clinic membership, on the other hand, had a strong association to all of the post-intervention implementation outcomes scores based on eta square coefficients ranging from .63 to .84. Consequently, clinic membership was included as a predictor alongside readiness in the second set of regression models. Table 6 presents the results of the regression analyses by implementation outcome. In summary, when included as the sole predictor, readiness levels accounted for between 44 and 68% (based on *R*^2^ values; adjusted *R*^2^ ranged from .42 to .66) of the variance in the implementation outcomes models. When clinic membership was added as a predictor alongside readiness levels, the implementation outcomes models resulted in *R*^2^ between .67 and .78 (with adjusted *R*^2^ ranging from .64 to .76). Beta coefficients were significant for both readiness levels and clinic membership in the acceptability, feasibility, and adoption models, but only clinic membership had a significant beta coefficient in the appropriateness model. Uniqueness indices indicated a fair amount of shared variance among predictors in all models, with readiness levels of particular importance for feasibility and intent to adopt, and clinic membership for appropriateness.

**Table 6:** Regression results

Early implementation outcome	Predictor(s)	***R***^**2**^	Adjusted ***R***^**2**^	***F***-statistic	Beta (*β*) coefficient (standardized)	***R***^**2**^ change
Acceptability	Readiness	0.55	0.53	28.39*	0.74*	N/A
	Readiness and clinic membership	0.72	0.70	28.64*	Readiness: 0.42* Clinic membership: 0.53*	Readiness: 0.11 Clinic membership: 0.17
Appropriateness	Readiness	0.44	0.42	13.38*	0.67*	N/A
	Readiness and clinic membership	0.77	0.74	35.83*	Readiness: 0.22 Clinic membership: 0.72*	Readiness: 0.03 Clinic membership: 0.32
Feasibility	Readiness	0.60	0.58	34.43*	0.77*	N/A
	Readiness and clinic membership	0.67	0.64	22.42*	Readiness: 0.56* Clinic membership: 0.34*	Readiness: 0.20 Clinic membership: 0.07
Intent to adopt	Readiness	0.68	0.66	47.89*	0.82*	N/A
	Readiness and clinic membership	0.78	0.76	39.85*	Readiness: 0.56* Clinic membership: 0.42*	Readiness: 0.19 Clinic membership: 0.11

Readiness as predictor, *F*(1, 23); readiness and clinic membership as predictors, *F*(2, 22)

*p < 0.05

## Discussion

Assessing and building readiness has been recognized as an essential strategy to ensure successful implementation. However, empirical studies investigating the relationship between use of a readiness intervention, readiness levels, and implementation outcomes are lacking. The purpose of this study was to (1) evaluate the effectiveness of a readiness intervention and (2) assess the role of readiness as a predictor of early implementation outcomes. To our knowledge, this study is the first attempt to empirically assess the relationship between readiness and implementation outcomes.

The study findings produced three key insights with theoretical and practical implications. First, *readiness to implement a new service accounted for 44 to 68% of the variance in the various early implementation outcomes*. Although preliminary, this finding aligns with previous claims suggesting that 50% of failed change efforts may be due to a lack of readiness [6, 9]. Even when accounting for clinic membership, levels of readiness remained a significant predictor of service acceptability, feasibility, and intent to adopt. Of note, similar trends were noted in both commitment and efficacy readiness scores. In other words, these early indicators of perceived implementation success are influenced by the extent to which the clinic team is able and committed to embrace this change. Consistent with previous claims [1–6], this result highlights the importance of readiness as an essential step in the pre-implementation process.

Second, it appears that the context in which the service is being implemented may be more important than readiness levels for predicting certain implementation outcomes. More specifically, appropriateness was significantly influenced by clinic membership, but not readiness, when both predictors were entered into the regression model. In other words, *compatibility of the service with clinic culture, patient care approach, target patient population, and existing workflows and operational processes may be more heavily dependent on the clinic than the clinic team’s levels of readiness*. This finding highlights the importance of organizational identity in predicting service fit. As an example, if the goal of a clinic is to treat a specific disease, integrating a service like CMM that is whole patient-centered may be too much of a cultural misalignment to be successful. This is not to say that the readiness intervention cannot be effective at increasing appropriateness. In fact, results across clinics supported that appropriateness was positively impacted by the readiness intervention. Rather, this finding indicates that, from an implementation practice perspective, focusing on enhancing fit as part of the readiness intervention might require more time and effort than is feasible. Some aspects of fit (e.g., clinic culture) may be difficult to change, others (e.g., workflows) may be more malleable. An alternative approach would be to determine service fit prior to a clinic engaging in the readiness process. This strategy would maximize the benefits of the readiness intervention for those clinics that have determined service fit (or at the very least concluded that enhancing fit was achievable). It would also expedite decisions not to implement a service for those low in appropriateness scores without the need to engage in a readiness process.

Conceptually, this second finding aligns with existing literature recommending that appropriateness be assessed prior to building readiness rather than be incorporated into the readiness intervention [6, 12]. Although the concept of fit has been identified as critical to implementation success [19, 30], few implementation frameworks make a conceptual distinction between fit and readiness [31]. This gap could be addressed more explicitly in existing implementation frameworks to legitimize the importance of fit as a precursor of readiness success.

Finally, *engaging in a readiness intervention is valuable in ways more complex than simply enhancing outcomes*. At first glance, it appears that the readiness intervention was effective at increasing readiness levels and positively influenced early implementation outcomes. However, upon closer examination, the direction of this association seemed to be dependent on the clinic participants worked in. While five of the clinics significantly increased their commitment and ability to implement CMM, readiness scores for two clinics trended downward. Similar patterns were observed for implementation feasibility, acceptability, and to some extent appropriateness, with the same two clinics also expressing the intent to not adopt CMM by the end of the readiness phase.

Qualitative data provided further insights into potential reasons for this result. Engaging in the readiness process helped participants deepen their understanding of the service and served to highlight the implications and practical realities of implementing CMM within their clinics. The differential impact of participating in a readiness intervention might therefore result from the realization that implementing a particular service may or may not be realistic within a given clinic environment.

This third insight carries conceptual implications for understanding and evaluating the impact of a readiness intervention. It may be worth broadening definitions of success and outcome metrics when assessing the impact of a readiness intervention. Success should not solely be evaluated based on increased readiness and positive trends in early implementation outcomes. For some, participating in a readiness intervention will lead to decreasing commitment and realization that gaps in capabilities preclude use of the service. Beyond preparing for implementation, the value of a readiness intervention may lie in the opportunity to systematically consider the practical implications associated with service delivery and make informed decisions about its adoption.

### Limitations

Although this study produces useful insights, it is not without limitations. As a preliminary exploratory study, the focus was on early implementation outcomes. The impact of readiness interventions needs to be investigated further by examining the implementation outcomes collected during different implementation stages. Ultimately, it is critical to determine whether engaging in a readiness process predicts implementation success, thereby increasing the likelihood of positive service outcomes. In addition, the generalizability of findings is limited due to a small purposive sample engaging in a specific readiness intervention to prepare for implementation of CMM. The results need to be further validated through additional studies focusing on other readiness interventions, different healthcare settings, and a wide range of services. Finally, it is important to note that the purpose of this study was not to test the R=MC^2^ heuristic that framed and guided the operationalization of the readiness intervention. Rather, the goal was to investigate whether the resulting readiness intervention was effective at influencing the commitment and ability to implement CMM and early implementation outcomes. Although common readiness concerns were addressed as a group, each clinic was free to select its own readiness goals and strategies to address any or all of the three R=MC^2^ components. Assessing the role of specific R=MC^2^ components in predicting implementation outcomes could be a potential direction for future research.

## Conclusion

In conclusion, this preliminary exploratory study supports the importance and value of readiness as an essential precursor of early implementation outcomes. Future studies could focus on replicating the current results using a range of readiness interventions across diverse healthcare contexts implementing different services; gaining additional insights into the associations between service appropriateness (fit), readiness, and implementation; and further understanding the evolution of the relationship between readiness and implementation outcomes over time. Continuing to build the empirical evidence base for readiness is critical to legitimizing its value as a precursor of successful implementation.

## Data Availability

The datasets used and/or analyzed during the current study are available from the corresponding author on reasonable request.

## Abbreviations

CMM: Comprehensive medication management
CMO: Center for Medication Optimization
CPP: Clinical practitioner pharmacist
DV: Dependent variable
IOQ: Implementation Outcomes Questionnaire
IV: Independent variable
MCDOP: Medical Center Department of Pharmacy
NP: Nurse practitioner
ORIC: Organizational Readiness for Implementing Change
PA: Physician assistant
RDS: Readiness Diagnostic Scale
VIF: Variance inflation factor

## Footnotes

1 The low sample size precluded any type of multi-level hierarchical or nested analyses, hence the inclusion of clinic membership as an independent variable of interest.
